# Biomarkers for enhancing the radiosensitivity of nasopharyngeal carcinoma

**DOI:** 10.7497/j.issn.2095-3941.2014.0015

**Published:** 2015-03

**Authors:** Wei Chen, Guo-Hua Hu

**Affiliations:** Department of Otorhinolaryngology, the First Affiliated Hospital of Chongqing Medical University, Chongqing 400016, China

**Keywords:** Nasopharyngeal carcinoma (NPC), radiotherapy, radiosensitization, biomarkers

## Abstract

Nasopharyngeal carcinoma (NPC) is a common head and neck malignancy. The incidence of NPC is higher in Southern China and Southeast Asia compared with Western countries. Given its high radiosensitivity, the standard treatment for NPC is radiotherapy. However, radioresistance remains a serious obstacle to successful treatment. Radioresistance can cause local recurrence and distant metastases in some patients after treatment by radiation. Thus, special emphasis has been given to the discovery of effective radiosensitizers. This review aims to discuss the biomarkers, classified according to the main mechanisms of radiosensitization, which can enhance the sensitivity of NPC cells to ionizing radiation.

## Introduction

Nasopharyngeal carcinoma (NPC) is a highly metastatic cancer that originates from the epithelial lining of the nasopharynx. NPC occurs mostly in the pharyngeal recess (fossa of Rosenmüller) and the eustachian tube opening in the nasopharynx^1^. Compared with other head and neck malignancies, NPC is unique in the aspects of epidemiology, pathology, and clinical manifestation. The etiology involves various factors, including genetic susceptibility, Epstein-Barr virus (EBV) infection, and exposure to chemical carcinogens^1–3^.

According to the histological classification of World Health Organization, NPC is divided into three types: keratinizing squamous cell carcinoma (type I), non-keratinizing squamous cell carcinoma (type II), and undifferentiated carcinomas (type III)^1^. In comparison with the differentiated NPC, undifferentiated NPC is more aggressive, with a higher incidence of distant metastasis^4^.

NPC exhibits a remarkable geographic distribution that the incidence in southern parts of China and Southeast Asia, usually belonging to types II and III, is higher than that in Western countries. In the West, NPC occurs sporadically and usually belongs to type I^1^.

Distinguished by its unique clinical and pathologic characteristics, NPC is highly radiosensitive. Compared with type I, types II and III have a higher response rate to ionizing radiation^4^. Thus, radiotherapy serves as a standard treatment that is effective to control the early tumor with good prognosis, achieving a 5-year overall survival of 90% and 84% for early stage I and IIA disease, respectively^5^. However, along with radiotherapy, radioresistance also exists in many cases. Moreover, radioresistance can cause locoregional recurrence and distant metastasis after radiotherapy in some patients, especially when the tumor is in the advanced stage (stage III or IV)^6^.

Therefore, for the relatively advanced stage, combined radio-chemotherapy may increase survival rate, which is 50%-70% as reported^6^. However, radioresistance still exists in these cases. Along with the damage of radiation to normal tissues, radioresistance remains a serious obstacle to successful treatment. Hence, methods of decreasing NPC radioresistance and enhancing radiosensitivity are urgently needed.

In this context, special emphasis is given to the exploitation of effective radiosensitizers according to the three main categories of radiosensitization mechanisms^7^.

## Biomarkers for enhancing radiosensitivity

### Repair inhibition

Cellular radiation repair is a type of ability that is required and vital for cells. Equipped with this ability, cells can correct radiation damage in various ways. First, cellular systems may prevent damage before it occurs. Second, cells may contain molecules that can restore damaged sites chemically. Third, different types of molecules and enzymatic systems can identify and repair damaged substrates through a set of complex and ordered reactions^7^. As follows, some biomarkers, participating in the repair of radiation damage, are related to radiosensitivity of NPC.

### NFBD1/MDC1

Nuclear factor with BRCA1 C-terminal (BRCT) domain 1/mediator of the DNA damage checkpoint protein 1 (NFBD1/MDC1) is one of the BRCT superfamily members that contains a forkhead-associated domain at the amino-terminus, two BRCT domains at carboxy-terminus, and central Pro-Ser-Thr repeat^8^. Previous studies showed that in response to DNA damage, the ataxia telangiectasia mutated (ATM) kinase catalyzes the phosphorylation of histone variant H2AX to recognize and marks the sites of damaged DNA^9^. NFBD1 then recruits repair factors including MRE11, RAD50, and NBS1 complex to the marked sites to form a nuclear foci, thus contributing to an increase in the fidelity of genomic integrity^8^. Furthermore, Lou *et al*.^10^ found that the depletion of endogenous NFBD1 can cause a complete loss of nuclear foci formation and induce genomic instability and DNA repair defects. This finding demonstrates that NFBD1 has a close relationship with the initiation and promotion of DNA repair process. *In vivo*, the generation of *MDC1* knockout mice had the characteristics of growth retardation, male infertility, defective class switching, and increased radiation sensitivity^10^. The expression level of *NFBD1* in NPC cells was absolutely high, and the suppression of *NFBD1* could increase the rate of apoptosis of NPC cells and the sensitivity to chemotherapy^11,12^. Recently, Wang *et al*.^13^ demonstrates that Lentivirus-mediated shRNA targeting *NFBD1* can steadily silence the expression of *NFBD1* gene and enhance the radiosensitivity of CNE-1 cells. These results may eventually contribute to the clinical investigation of prediction markers and prognostic factors, as well to the development of radiosensitizing molecules for therapeutic use.

### Cell cycle redistribution

Radiosensitivity of the cells varies according to their positions in the cell cycle. Cells in G_2_/M are approximately 3-fold more sensitive to radiation than cells in late S-phase/early G_1_, but the exact cause of variation is currently not completely known^14^. Thus, agents that can block the progression of a cell cycle in a radiosensitive phase may induce significant radiosensitization.

#### COX-2

Cyclooxygenase or prostaglandin endoperoxidase synthase (COX) is an enzyme that participates in the formation of prostaglandins (PGs) and is also recognized as an important chemical mediator for inflammation. COX has two isoforms: COX-1 and COX-2. COX-2, not usually expressed in most normal tissues, has a close relationship with the synthesis of PGs in inflamed and neoplastic tissues^15^. COX-2 overexpression has been reported in most cancers, such as esophageal, gastric, pancreatic, colorectal, lung, and breast cancers^16–18^, suggesting that COX-2 overexpression has a close relationship with cancer progression^15^. Some preclinical studies have investigated the role of COX-2 in carcinogenesis^19^. In these studies, upregulation of COX-2 can regulate angiogenesis, resist apoptosis, and influence cell proliferation successfully through induction of vascular endothelial growth factor (VEGF) and translocation of HIF-1α protein, increase of the expression of proapoptotic Bcl-2 proteins, inhibition of cytochrome c released from mitochondria, and control of G_1_ and S phase cyclins^20^. Thus, the selective COX-2 inhibitors can be regarded as anticancer agents. Recently, a preclinical study showed that high *COX-2* expression was found in more than 70% of NPC, and these tumors had earlier regional lymph node metastasis, distant metastasis, and poor responses to chemotherapy, as well as a lower 5-year survival rate compared with low *COX-2* expression or negative tumors^21^. Meanwhile, an *in vitro* study demonstrated that adding the selective COX-2 inhibitor NS-398 in NPC cells could increase the radiation-induced cell death^22^. Furthermore, Sun *et al*.^23^ showed that *COX-2* overexpressing proteins in NPC cells have radioprotective effects, and silence of the expression of *COX-2* can reduce radiation-induced damage repair, thereby enhancing cellular radiosensitivity; this finding lays a theoretical foundation on new gene-radiation combined therapy for NPC. The underlying mechanism may be attributable to the G_2_-M cell phase arrest and enhancement of cell apoptosis^24^.

#### *GP96* or *GDF-15*

The 96 kDa glycoprotein (GP96, also known as GPR94) is an endoplasmic reticulum resident protein that belongs to the heat shock protein 90 family. This glycoprotein is involved in the package and transport of membrane-bound oligomeric proteins, such as immunoglobulins, epidermal growth factor receptor (EGFR), and integrins. It can also induce a variety of immune responses, including antitumor immune responses and the productions of antigen-processing peptides, to collect MHC class I molecules^25^.

Growth differentiation factor 15 (GDF-15) is a member of the transforming growth factor β superfamily. In response to DNA damage, GDF-15 is also a significant downstream mediator. Moreover, high expression of GDF-15 can induce apoptosis and G_1_ cell cycle arrest^26^. Chang *et al*.^27^ used siRNA to suppress the expression of *GP96* and *GDF-15* in NPC-radioresistant cells, which demonstrated that silence of these genes could cause cell growth delay, G_2_-M cell cycle arrest, and a reduction of clonogenic survival. They also verified that knockdown of the genes could increase the radiosensitivity of NPC cells^27^. Although further clinical studies are needed, these results may eventually contribute to the development of novel radiosensitizing therapeutics.

#### GnT-V

Protein glycosylation, a type of post-translational modification, is vital for the glycoprotein, which can affect cell growth, differentiation, and tumor metastasis^28^. The glycosyltransferase, located in the Golgi apparatus, plays an important role in protein glycosylation. The glycosyltransferase contains at least six N-acetylglucosaminyltransferases (GnTs), defined as GnT-I-VI. N-glycosyltransferase-V (GnT-V) is recognized as a primary member of the glycosyltransferase family, catalyzing the formation of β1, 6 GlcNAc branching structures. Many studies have shown that upregulation of the β1, 6 GlcNAc branched N-glycans structure is related to malignant transformation through various ways, such as inhibiting cell apoptosis and enhancing cell proliferation^29^. Previous studies have shown that GnT-V plays an important role in malignant tumors. Recently, Wei *et al*.^30^ demonstrated that downregulation of GnT-V inhibited NPC cell line CNE-2 clonogenic survival after radiation *in vitro*. Another study investigated the role of GnT-V on the radiosensitivity of NPC cells and its underlying mechanisms. These results showed that downregulation of GnT-V in NPC enhanced radiosensitivity *in vitro* and *in vivo*. The underlying mechanisms may be linked to the cell cycle G_2_-M arrest and the reduction of Bcl-2/Bax ratio. GnT-V may be a potential target for predicting NPC response to radiotherapy^31^.

#### Jab1/CSN5

C-Jun activation domain-binding protein-1 (Jab1) is a modulator of intracellular signaling. Through its existence as the fifth component of the constitutive photomorphogenic-9 signalosome (CSN5), Jab1/CSN5 can regulate cellular proliferation and apoptosis by functionally deactivating some key negative regulatory proteins and tumor suppressors, such as p53 and p27^32^. In previous studies, overexpression of *Jab1* was found to be correlated with poor prognosis in some tumor types, such as pancreatic adenocarcinomas^33^, breast cancers^34^, and NPC^35^. Other previous studies had found that Jab1 played an important role in the pathologenesis and radioresistance in NPC^36^.

Curcumin is a common chemopreventive agent. Although it plays an important role in anticancer activities in tumor cells^37^, many previous studies showed that curcumin was limited in its application in anticancer therapy because of its poor bioavailability, rapid metabolism, and instability under several physiologic conditions^38^. Thus, analogues of curcumin, which have increased anticancer activity and similar safety profiles as curcumin, have been developed in recent years^39,40^. Pan *et al.*^41^ used T83 (a new 4-arylidene curcumin analogue) to inhibit the expression of *Jab1* in NPC cells, demonstrating that inhibition of Jab1 could reduce tumor cell growth, induce G_2_/M arrest, and increase tumor cell apoptosis, thus enhancing the sensitivities of NPC cells to radiotherapy. This study verified T83 as a potential targeted therapy, which may increase the radiosensitivities of NPC cells, providing a promising and effective treatment for NPC patients.

### Increased initial damage

Through production of free radical species and direct ionization of target molecules, ionizing radiation can cause cellular damage. DNA is the standard target of ionizing radiation damage, and radiation induced double-strand DNA breaks are fatal. Although we do not completely know the exact type of radiation-induced damage, we can use the agents that could cause more initial damage to cellular targets to achieve an increasing cytotoxic effect of radiation.

#### DNA topoisomerase I

DNA topoisomerases, first discovered in 1971, are important enzymes that can regulate the transformation of DNA topological isomer. DNA topoisomerases have two types: DNA topoisomerase I and DNA topoisomerase II^42^. Topoisomerase I (Top I), which is ATP-independent, catalyzes the relaxation of superhelical DNA by cutting off a single chain, allowing the intact chain through the broken chain (Top IA) or leaving the broken chain free to rotate around the intact chain (Top IB). Topoisomerase II (Top II), which is ATP-dependent, mediates the generation of breaks in both chains of the DNA duplex^43^. Both topoisomerase I and II play important roles in many aspects of DNA metabolism, such as DNA replication and DNA transcription, especially the topoisomerase I, which is very essential for the maintenance of the genomic stability^42^. Furthermore, drugs that interfere with topoisomerase I-mediated cleavage rejoining of DNA chains can be widely used in the treatment of cancer^44^. These drugs have also been regarded as effective radiosensitizers when used before radiotherapy, including derivatives of camptothecin^45^. Topotecan (TPT) is a derivative of camptothecin, which can inhibit topoisomerase I in S phase cells. TPT has been suggested to be effective in the radiotherapy of NPC. Zhang *et al.*^46^ established a xenografted human NPC model in nude mice and investigated the synergistic effect of TPT and chronoradiotherapy. The results showed that TPT has a radiosensitizing effect on xenografted human NPC. More importantly, TPT has a circadian dependence, thus contributing to further optimization of therapeutic schedules for NPC. Moreover, the potential mechanism of chronomodulated radiosensitization of TPT may be related to the circadian rhythm of tumor hypoxia and the radiation reluctance of cells in S phase, causing G_2_/M phase arrest.

#### Nitric oxide synthases

NO is a gaseous molecule that has two types: enzyme-dependent NO and enzyme-independent NO. In human cells, NO is enzyme-dependent and comes from L-arginine catalyzed by NO synthases (NOS). NO plays a very important role in cell signaling pathway through cell-mediated immune responses, cytotoxicity, formation of guanylate cyclase, and cyclic guanosine monophosphate^47^. Previous studies showed that NO is a hypoxic cell radiosensitizer when combined with radiotherapy^48^. Recent evidence showed that NO is an initiator of apoptotic signals due to its ability to form peroxynitrite, which can activate oxidative stress-induced apoptosis^49^. Stimulated by cytokines, inducible NOS (iNOS) can produce much NO. Thus, increased iNOS is associated with increased apoptosis. To explore the function of iNOS in NPC, Jayasurya *et al*.^50^ demonstrated that low *iNOS* expression comes with high incidence of tumor local recurrence and metastasis, probably because of decreased apoptosis. In addition, NPC cells with high *iNOS* expressions may be more sensitive to radiotherapy.

### Others

The mechanisms of the following biomarkers do not belong to the three main categories or are not completely known right now.

#### *EGFR* and *IGF-1R*

EGFR and insulin-like growth factor-1 receptor (IGF-1R) are key members of the tyrosine kinase receptor family, whose ligands are respectively epidermal growth factor and insulin-like growth factor-1 (IGF-1). The combinations of both receptor and ligand are involved in the development of malignant tumor through modulation of tumor cell cycles, apoptosis, and interactions with the surrounding environment^51^. Moreover, IGF-1R plays a crucial role in the generation of drug resistance during anticancer therapy and also in the proliferation and transformation of tumor cells. Meanwhile, EGFR significantly contributes to promotion of cell proliferation^52^. Early experiments showed that combining short hairpin RNA segments to *IGF-1* and *EGFRs* via plasmid or virus transfection could significantly inhibit NPC cell growth, induce apoptosis, and increase chemotherapy sensitivity *in vitro*^53^. Meanwhile, another study found that overexpression of *EGFR* and *IGF-1R* in advanced and recurrent NPC tissues affected the radiosensitivity of NPC cells^54^. Recently, Liu *et al*.^55^ verified that the dual-silencing of *IGF-1R* and *EGFR*, which are capable to decrease the expression of NPC cyclins and to block cell cycles, may promote the radiosensitivities and apotosis of NPC cells.

#### VEGF

VEGF, earlier known as vascular permeability factor, is a specific heparin-binding growth factor of vascular endothelial cells that can induce angiogenesis to support the growth of both normal and tumor tissues *in vivo*. As it is very essential for the progressive enlargement of malignant tumor, the function of VEGF in cancer treatment has been widely studied. Some studies found that VEGF made a great contribution to radioresistance in cancer radiotherapy both *in vitro* and *in vivo*. Those results suggested that targeting VEGF may inhibit cancer cell growth, decrease radioresistance, and prevent metastasis^56^.

Angiotensin II (Ang II) is an important part of the renin–angiotensin-aldosterone system, which can regulate the expression of vasoconstriction, vasopressin, and aldosterone; induce angiogenesis by upregulating VEGF; and promote cell proliferation. In the circulatory system, almost all the physiological functions of Ang II are generated by stimulating the angiotensin II type 1 receptors (AT1R)^57^. Valsartan is a type of AT1R antagonist that can cut off the binding of Ang II to AT1R to reduce the production of VEGF. Previous studies reported that NPC cells could produce VEGF^58^, and its expression had a close relationship with the clinical stage^59^ and the prognosis of NPC^60^. In exploring whether valsartan plays a role in the treatment of NPC, Wang *et al*.^61^ revealed that the expression of AT1R was very high in NPC cells, and AT1R blocker valsartan could suppress the secretion of VEGF in NPC cells, thus inhibiting cancer cell growth. All these results implied that AT1R blocker valsartan could increase the radiosensitivity of NPC cells. Meanwhile, Zhou *et al*.^62^ used RNA interference (RNAi) technology to block *VEGF* expression of NPC cells, thus suppressing the proliferation and migration of NPC cells, inducing tumor apoptosis, and increasing the therapeutic efficacy of ionizing radiation. Therefore, methods to decrease VEGF in NPC cells may have great potential as novel therapeutic strategies against NPC.

#### LMP-1

EBV, a prototype gamma herpes virus that infects the great majority of the population worldwide, has been implicated in the pathogenesis of several human malignancies including lymphoproliferative malignancies, such as Burkitt’s and Hodgkin’s lymphomas, as well as epithelial tumors, such as NPC and gastric carcinoma. EBV infection in NPC is classified as latency type II, which is characterized by the expression of a limited series of latent genes, including EBV nuclear antigen-1, latent membrane protein-1 (LMP1), LMP2, and EBV early RNA^63^. Considering its oncogenic potential, LMP1 is speculated to be an essential factor in the tumorigenesis of NPC^64^. The LMP1 protein is an integral membrane protein and can be subdivided into three domains: a short intracellular N terminus, which orientates the LMP1 protein to the plasma membrane; six hydrophobic transmembrane domains, which are involved in self-aggregation and oligomerization^65^; and an intracellular carboxyl-terminal activating region, which activates most of LMP1’s signaling activities^66^ and induces a variety of downstream pathological changes in cell proliferation, anti-apoptosis, and metastasis^67,68^. Therefore, Yang *et al*.^69^ successfully obtained a phosphorothioate-modified “10-23” DNAzyme, named DZ1, which could downregulate the expression of *LMP1* in NPC cells, to explore the potential use of DNAzymes for the new treatment of NPC. All results demonstrated that low expression of *LMP1* in NPC cells could inhibit cell proliferation and metastasis, promote apoptosis, and enhance radiosensitivity of NPC. In addition, the potential mechanism is through the interferences of signal pathways, which are launched by LMP1 including NF-κB, AP-1, and STAT3 signal pathways^69^. Recently, Ma *et al*.^70^ confirmed that LMP1 could regulate the expression of *ATM* via the NF-κB pathways, thus resulting in the change of radiosensitivity in NPC cells. Moreover, Xiao *et al*.^71^ used a metabolomics approach to investigate EBV-modulated metabolic changes, indicating that there is a close relationship between EBV and glycolysis in NPC. Significantly, LMP1 plays a key role in the reprogramming of EBV-mediated glycolysis in NPC cells, and anti-glycolytic therapy can effectively enhance the sensitivity of *LMP1*-overexpressing NPC cells to radiation. Thus, anti-glycolytic therapy may be a novel therapeutic strategy for NPC patients^71^.

#### Protein kinase CK2

Protein kinase CK2 (formerly known as casein kinase 2 or II) is a common serine/threonine protein kinase that is localized in the cytoplasmic and nuclear compartments of the cell. Protein kinase CK2 contains two regulatory (28-kDaβ) subunits and two catalytic (42-kDaα and 38-kDaα') subunits, forming the heterotetrameric configurations α2β2, αα'β2, and α'2β2. The role of CK2, which is multifunctional, in normal and abnormal cell growth, proliferation, and apoptosis has been recognized for a long time. CK2 exerts its anti-apoptotic effects through the phosphorylation of a series factors, and inhibition of CK2 can induce apoptosis and promote tumor cell proliferation^72^. Furthermore, Liu *et al*.^73^ used RNAi technique to downregulate the *protein kinase CK2α* expression in NPC cells and demonstrated that *CK2α* knockdown significantly decreased the clonogenic activity and increased the radiosensitivity of the NPC cells. Hence, suppression of the expression of *CK2* may be a potential therapeutic approach to NPC.

#### Bcl-2

*Bcl-2* gene (also known as B-cell lymphoma/leukemia gene-2) is one of the strongest genes that have an anti-apoptotic effect and is recognized as a human longevity gene^74^. *Bcl-2* gene can inhibit many apoptotic signal-induced apoptosis, promote cell survival, and disorder regulatory mechanism of apoptosis *in vivo*; this gene is found expressed in a variety of malignancies, such as NPC, lung cancer, and cervical cancer^75^. Previously, a study found that *Bcl-2* gene played a significant role in the promotion of tumor invasion and metastasis, and its evolvement had a close relationship with the progression of NPC^76^. Other studies demonstrated that Bcl-2 and Bcl-2/bax could be used as important indicators of radiation sensitivity in treating NPC cells^77^. Furthermore, Tian *et al*.^78^ successfully suppressed the expression of Bcl-2 protein by using RNAi technology, thus contributing to an increase of the radiosensitivity of NPC cells. All results suggested that *Bcl-2* may be a potential indicator for predicting the responses of NPC cells to radiotherapy.

#### Notch signaling pathway

Notch gene, first found in 1917 by Morgan^79^, is involved in the Notch signaling pathway, which is essential to determine cell fate, maintain the stability of stem cell, and regulate the pattern formation. In addition, its dysfunction is related to various defects of developments and human pathologies, including tumorigenesis^80^. Notch signaling pathway is found highly expressed in many malignant tumors, such as breast cancer^81^, glioma^82^, and NPC^83^. This pathway can be activated by EBV-coded latent membrane protein 2A, which is also an important factor to NPC oncogenesis^84^. Thus, Notch signaling pathway plays a vital role in NPC tumorigenesis.

γ-secretase, a type of proteolytic enzyme, is very essential for the Notch signaling pathway, which can release the Notch intracellular domain. Given that Notch signaling pathway contributes to the progression of NPC, γ-secretase inhibitors (GSIs) that block Notch signaling pathway may achieve better effects when combined with radiotherapy^85^. Yu *et al*.^86^ investigated the effect of one GSI (N-[(3,5-difluorophenyl)acetyl]-L-alanyl-2-phenyl]glycine-1,1-dimethylethylester, DAPT) combined with radiotherapy. This study demonstrated that downregulation of Notch signaling pathway could increase the radiosensitivity of NPC cells, thus indicating GSIs as radiosensitizers for NPC treatment^86^.

## Conclusion

NPC is a common head and neck cancer. The incidence rate is higher in southern China and Southeast Asia than in Western countries. Given its high radiosensitivity, radiotherapy is considered the primary treatment for NPC. However, radiotherapy is challenging for NPC because of the radiosensitive structures that surround the nasopharynx, including the brain stem, spinal cord, pituitary-hypothalamic axis, temporal lobes, eyes, ears, and parotid glands^87^. In addition, radiotherapy often brings adverse effects, such as xerostomia and temporal lobe necrosis. Many NPCs are resistant to radiation, which brings difficulties to treatment^87^. To improve the efficacy of radiotherapy, the use of radiosensitizers combined with ionizing radiation is beneficial.

The concept of radiosensitizers was first proposed in 1958. All the chemical and biological methods that can affect the sensitivity to radiation are named radiosensitizers, including chemotherapeutic drugs and molecular targeted agents^88,89^. Chemotherapeutics, such as 5-fluorouracil, platinum analogs, and DNA topoisomerase I-targeting drugs, are commonly used when combined with radiotherapy and can achieve better local-regional therapeutic effects^90^. Molecular targeted agents that target DNA or not are also helpful to radiation therapy, such as *EGFR* blockers and *COX-2* inhibitors^89^. In this article, we have summarized some of the biomarkers, classified according to the main mechanisms of radiosensitization, to enhance the responsiveness of NPC cells to radiation treatment (**Table 1**).

**Table 1:** Biomarkers for enhancing the radiosensitivity of nasopharyngeal carcinoma (NPC)

Biomarkers	Potential mechanism of radiosensitization of NPC	Methods	References
*NFBD-1*	Induction of a complete loss of nuclear foci formation, DNA repair defects, and genomic instability	Use of Lentivirus-mediated shRNA targeting NFBD 1 to silence the expression of *NFBD 1* gene in CNE-1 cells	^11–13^
*COX-2*	Reduction of G_2_/M phase arrest in order not to be fully repaired after receiving radiation	Use of shRNAmir lentiviral vector to silence the expression of *COX-2* gene	^22–24^
*GP96 or GDF-15*	Elevation of the proportion of the cells in radiosensitive G_2_-M phase	Use of siRNA to knockdown the *gp96* and *GDF-15* in NPC-radioresistant cells	^26,27^
*GnT-V*	Arrest of cell cycle G_2_-M and reduction of Bcl-2/Bax ratio	Use of Lipofectamine 2000 to transfect the plasmid of antisense GnT-V cDNA into CNE-2 cells	^30,31^
*Jab1/CSN5*	Arrest of cell cycle G_2_-M	Use of T83 (a new 4-arylidene curcumin analogue) to inhibit the expression of *Jab1* in NPC cells	^39–41^
DNA topoisomerase I	Relationship with the circadian rhythm of tumor hypoxia and G_2_/M phase arrest	Administration of Topotecan (DNA topoisomerase I-targeted drug) into xenografted human NPC model through peritoneal injection	^45,46^
Nitric oxide synthases (NOS)	Direct cellular toxicity or interaction with NO reactive species that increase apoptosis	Use of terminal deoxynucleotidyl transferase-mediated dUTP-biotin nick end labeling assay to detect the expression of inducible nitric oxide synthase	^50^
*EGFR and IGF-1R*	Decrease of the expression of NPC cyclins and blocking cell cycles	Use of plasmid or virus transfection to combine short hairpin RNA segments to insulin-like growth factor 1 receptor and to epidermal growth factor receptor	^54,55^
*VEGF*	Reduction of tumor angiogenesis that inhibit cancer cell growth, prevent metastasis, and decrease resistance to therapy	Use of valsartan (an AT1R antagonist that can inhibit *VEGF* expression and secretion in NPC cells)	^61,62^
*LMP-1*	Interference of signal pathways, which are abnormally activated by LMP1, including NF-κB, AP-1, and STAT3 signal pathways	Use of phosphorothioate-modified “10–23” DNAzyme, namely, DZ1, to downregulate the expression of *LMP1* in NPC cells	^70,71^
*Protein kinase CK2*	It exerts its anti-apoptotic effects through the phosphorylation of a series of factors, but the mechanism of radiosensitization is unknown right now	Use of RNAi technique to downregulate the *protein kinase CK2α* expression in NPC cells	^73^
*Bcl-2*	Inhibition of many apoptotic signals induced apoptosis, promotion of cell survival, and disorder of regulatory mechanism of apoptosis *in vivo*	Use of RNAi technology to reduce the expression of Bcl-2 protein	^76–78^
Notch signaling pathway	Decrease of the proportion of cancer stem cells and inhibition of tumor growth	Use of γ-secretase inhibitors to inhibit Notch signaling	^80,85,86^

As described above, certain useful biomarkers can be silenced in NPC radiotherapy to enhance radiosensitivity. Meanwhile, to modify the expression of unwanted molecular biomarkers, short nucleic acid-based cancer therapeutics containing short interfering RNAs, antisense oligonucleotides, DNAzymes, and ribozymes were employed^91^. In addition, some agents can be used to silence or inhibit the expression of unwanted biomarkers, including topotecan, valsartan, and analogues of curcumin. Targeting the biomarkers to enhance the radiosensitivity of NPC is very promising and practicable. However, some problems need to be resolved. First, most of the candidate biomarkers are examined only in the molecular level studies and are not yet evaluated in large population clinical trials. Second, the mechanisms of some biomarkers leading to radiosensitization of NPC are not yet completely known. Third, whether some of the biomarkers are specific only to NPC or are common in other cancers is unclear. Hence, large cohort clinical trials are needed to verify the feasibility, availability, and safety of biomarkers to enhance the radiosensitivity of NPC cells.

## References

[1] Wei WI, Sham JS (2005). Nasopharyngeal carcinoma. Lancet.

[2] Tao Q, Chan AT (2007). Nasopharyngeal carcinoma: molecular pathogenesis and therapeutic developments. Expert Rev Mol Med.

[3] Lo KW, Chung GT, To KF (2012). Deciphering the molecular genetic basis of NPC through molecular, cytogenetic, and epigenetic approaches. Semin Cancer Biol.

[4] Barnett GC, West CM, Dunning AM, Elliott RM, Coles CE, Pharoah PD (2009). Normal tissue reactions to radiotherapy: towards tailoring treatment dose by genotype. Nat Rev Cancer.

[5] Lee AW, Sze WM, Au JS, Leung SF, Leung TW, Chua DT (2005). Treatment results for nasopharyngeal carcinoma in the modern era: the Hong Kong experience. Int J Radiat Oncol Biol Phys.

[6] Rottey S, Madani I, Deron P, Van Belle S (2011). Modern treatment for nasopharyngeal carcinoma: current status and prospects. Curr Opin Oncol.

[7] Poggi MM, Coleman CN, Mitchell JB (2001). Sensitizers and protectors of radiation and chemotherapy. Curr Probl Cancer.

[8] Stewart GS, Wang B, Bignell CR, Taylor AM, Elledge SJ (2003). MDC1 is a mediator of the mammalian DNA damage checkpoint. Nature.

[9] Fernandez-Capetillo O, Chen HT, Celeste A, Ward I, Romanienko PJ, Morales JC (2002). DNA damage-induced G2-M checkpoint activation by histone H2AX and 53BP1. Nat Cell Biol.

[10] Lou Z, Minter-Dykhouse K, Franco S, Gostissa M, Rivera MA, Celeste A (2006). MDC1 maintains genomic stability by participating in the amplification of ATM-dependent DNA damage signals. Mol Cell.

[11] Liu DD, Hu GH, Liu XL, Bu YQ (2012). Effect of siRNA-mediated NFBD1 depletion on chemosensitivity in nasopharyngeal carcinoma cells. J Chongqing Med Univ.

[12] Qiu ZL, Zeng Q, Liu XL, Hu GH (2013). Expressions of NFBD1 in nasopharyngeal carcinoma and their clinical significances. J Chongqing Med Univ.

[13] Wang ZH, Zeng Q, Chen T, Liao K, Bu YQ, Hong SL (2014). Effect of NFBD1 gene silencing by RNA interference on radiosensitivity of nasopharyngeal carcinoma CNE-1 cells. Tumor.

[14] Norbury CJ, Hickson ID (2001). Cellular responses to DNA damage. Annu Rev Pharmacol Toxicol.

[15] Ulrich CM, Bigler J, Potter JD (2006). Non-steroidal anti-inflammatory drugs for cancer prevention: promise, perils and pharmacogenetics. Nat Rev Cancer.

[16] Wang D, Dubois RN (2004). Cyclooxygenase-2: a potential target in breast cancer. Semin Oncol.

[17] Hill R, Li Y, Tran LM, Dry S, Calvopina JH, Garcia A (2012). Cell intrinsic role of COX-2 in pancreatic cancer development. Mol Cancer Ther.

[18] Zhao Y, Hao Y, Ji H, Fang Y, Guo Y, Sha W (2010). Combination effects of salvianolic acid B with low-dose celecoxib on inhibition of head and neck squamous cell carcinoma growth in vitro and in vivo. Cancer Prev Res (Phila).

[19] Mutter R, Lu B, Carbone DP, Csiki I, Moretti L, Johnson DH (2009). A phase II study of celecoxib in combination with paclitaxel, carboplatin, and radiotherapy for patients with inoperable stage IIIA/B non-small cell lung cancer. Clin Cancer Res.

[20] Shin YK, Park JS, Kim HS, Jun HJ, Kim GE, Suh CO (2005). Radiosensitivity enhancement by celecoxib, a cyclooxygenase (COX)-2 selective inhibitor, via COX-2-dependent cell cycle regulation on human cancer cells expressing differential COX-2 levels. Cancer Res.

[21] Tan KB, Putti TC (2005). Cyclooxygenase 2 expression in nasopharyngeal carcinoma: immunohistochemical findings and potential implications. J Clin Pathol.

[22] Chen WC, McBride WH, Chen SM, Lee KF, Hwang TZ, Jung SM (2005). Prediction of poor survival by cyclooxygenase-2 in patients with T4 nasopharyngeal cancer treated by radiation therapy: clinical and in vitro studies. Head Neck.

[23] Sun QQ, Liu X, Liu Y, Wang L, Peng XY, Zeng FF (2011). Effect of shRNAmir lentiviral vector-mediated COX-2 silencing on the radiosensitivity of nasopharyngeal carcinoma cells. Nan Fang Yi Ke Da Xue Xue Bao.

[24] Zhang SX, Qiu QH, Chen WB, Liang CH, Huang B (2014). Celecoxib enhances radiosensitivity via induction of G_2_-M phase arrest and apoptosis in nasopharyngeal carcinoma. Cell Physiol Biochem.

[25] Srivastava P (2002). Interaction of heat shock proteins with peptides and antigen presenting cells: chaperoning of the innate and adaptive immune responses. Annu Rev Immunol.

[26] Li PX, Wong J, Ayed A, Ngo D, Brade AM, Arrowsmith C (2000). Placental transforming growth factor-beta is a downstream mediator of the growth arrest and apoptotic response of tumor cells to DNA damage and p53 overexpression. J Biol Chem.

[27] Chang JT, Chan SH, Lin CY, Lin TY, Wang HM, Liao CT (2007). Differentially expressed genes in radioresistant nasopharyngeal cancer cells: gp96 and GDF15. Mol Cancer Ther.

[28] Ohtsubo K, Marth JD (2006). Glycosylation in cellular mechanisms of health and disease. Cell.

[29] Przybyło M, Martuszewska D, Pocheć E, Hoja-Łukowicz D, Lityńska A (2007). Identification of proteins bearing beta1-6 branched N-glycans in human melanoma cell lines from different progression stages by tandem mass spectrometry analysis. Biochim Biophys Acta.

[30] Wei T, Li Y, Zhuo E, Zhu W, Meng H, Zhang J (2011). Down-regulation of GnT-V inhibits nasopharyngeal carcinoma cell CNE-2 malignancy in vitro and in vivo. Cancer Lett.

[31] Zhuo E, He J, Wei T, Zhu W, Meng H, Li Y (2012). Down-regulation of GnT-V enhances nasopharyngeal carcinoma cell CNE-2 radiosensitivity in vitro and in vivo. Biochem Biophys Res Commun.

[32] Shackleford TJ, Claret FX (2010). JAB1/CSN5: a new player in cell cycle control and cancer. Cell Div.

[33] Kouvaraki MA, Korapati AL, Rassidakis GZ, Tian L, Zhang Q, Chiao P (2006). Potential role of Jun activation domain-binding protein 1 as a negative regulator of p27kip1 in pancreatic adenocarcinoma. Cancer Res.

[34] Kouvaraki MA, Rassidakis GZ, Tian L, Kumar R, Kittas C, Claret FX (2003). Jun activation domain-binding protein 1 expression in breast cancer inversely correlates with the cell cycle inhibitor p27(Kip1). Cancer Res.

[35] Pan Y, Zhang Q, Tian L, Wang X, Fan X, Zhang H (2012). Jab1/CSN5 negatively regulates p27 and plays a role in the pathogenesis of nasopharyngeal carcinoma. Cancer Res.

[36] Pan Y, Zhang Q, Atsaves V, Yang H, Claret FX (2013). Suppression of Jab1/CSN5 induces radio- and chemo-sensitivity in nasopharyngeal carcinoma through changes to the DNA damage and repair pathways. Oncogene.

[37] Ji JL, Huang XF, Zhu HL (2012). Curcumin and its formulations: potential anti-cancer agents. Anticancer Agents Med Chem.

[38] Anand P, Kunnumakkara AB, Newman RA, Aggarwal BB (2007). Bioavailability of curcumin: problems and promises. Mol Pharm.

[39] Debata PR, Castellanos MR, Fata JE, Baggett S, Rajupet S, Szerszen A (2013). A novel curcumin-based vaginal cream Vacurin selectively eliminates apposed human cervical cancer cells. Gynecol Oncol.

[40] Manohar S, Khan SI, Kandi SK, Raj K, Sun G, Yang X (2013). Synthesis, antimalarial activity and cytotoxic potential of new monocarbonyl analogues of curcumin. Bioorg Med Chem Lett.

[41] Pan Y, Wang M, Bu X, Zuo Y, Wang S, Wang D (2013). Curcumin analogue T83 exhibits potent antitumor activity and induces radiosensitivity through inactivation of Jab1 in nasopharyngeal carcinoma. BMC Cancer.

[42] Chen SH, Chan NL, Hsieh TS (2013). New mechanistic and functional insights into DNA topoisomerases. Annu Rev Biochem.

[43] Koster DA, Croquette V, Dekker C, Shuman S, Dekker NH (2005). Friction and torque govern the relaxation of DNA supercoils by eukaryotic topoisomerase IB. Nature.

[44] Staker BL, Feese MD, Cushman M, Pommier Y, Zembower D, Stewart L (2005). Structures of three classes of anticancer agents bound to the human topoisomerase I-DNA covalent complex. J Med Chem.

[45] Chen AY, Chou R, Shih SJ, Lau D, Gandara D (2004). Enhancement of radiotherapy with DNA topoisomerase I-targeted drugs. Crit Rev Oncol Hematol.

[46] Zhang Y, Chen X, Ren P, Su Z, Cao H, Zhou J (2013). Synergistic effect of combination topotecan and chronomodulated radiation therapy on xenografted human nasopharyngeal carcinoma. Int J Radiat Oncol Biol Phys.

[47] Carreras MC, Poderoso JJ (2007). Mitochondrial nitric oxide in the signaling of cell integrated responses. Am J Physiol Cell Physiol.

[48] De Ridder M, Van Esch G, Engels B, Verovski V, Storme G (2008). Hypoxic tumor cell radiosensitization: role of the iNOS/NO pathway. Bull Cancer.

[49] Shigyo H, Nonaka S, Katada A, Bandoh N, Ogino T, Katayama A (2007). Inducible nitric oxide synthase expression in various laryngeal lesions in relation to carcinogenesis, angiogenesis, and patients’ prognosis. Acta Otolaryngol.

[50] Jayasurya A, Dheen ST, Yap WM, Tan NG, Ng YK, Bay BH (2003). Inducible nitric oxide synthase and bcl-2 expression in nasopharyngeal cancer: correlation with outcome of patients after radiotherapy. Int J Radiat Oncol Biol Phys.

[51] Lorch JH, Posner MR, Wirth LJ, Haddad RI (2009). Seeking alternative biological therapies: the future of targeted molecular treatment. Oral Oncol.

[52] Dziadziuszko R, Merrick DT, Witta SE, Mendoza AD, Szostakiewicz B, Szymanowska A (2010). Insulin-like growth factor receptor 1 (IGF1R) gene copy number is associated with survival in operable non-small-cell lung cancer: a comparison between IGF1R fluorescent in situ hybridization, protein expression, and mRNA expression. J Clin Oncol.

[53] Yuan YL, Zhou XH, Song J, Qiu XP, Li J, Ye LF (2008). Dual silencing of type 1 insulin-like growth factor and epidermal growth factor receptors to induce apoptosis of nasopharyngeal cancer cells. J Laryngol Otol.

[54] Yuan Y, Zhou X, Song J, Qiu X, Li J, Ye L (2008). Expression and clinical significance of epidermal growth factor receptor and type 1 insulin-like growth factor receptor in nasopharyngeal carcinoma. Ann Otol Rhinol Laryngol.

[55] Liu GL, Yuan YL, Liu YJ, Zhou XH (2012). Dual Silencing of Insulin-Like Growth Factor-1 and Epidermal Growth Factor Receptors to Induce the Radioactivity of Nasopharyngeal Cancer Cells. Med J Wuhan Univ.

[56] Ye WJ, Min HQ, Cao XP, Chen KT, Hou JH, Li Y (2006). Correlations of biomolecular markers, such as P53 protein and vascular endothelial growth factor, to radiosensitivity of nasopharyngeal carcinoma. Ai Zheng.

[57] Mehta PK, Griendling KK (2007). Angiotensin II cell signaling: physiological and pathological effects in the cardiovascular system. Am J Physiol Cell Physiol.

[58] Yang HF, Tang WP (2003). Vascular endothelial growth factor gene expression in nasopharyngeal carcinoma cell line induced by hypoxia in vitro. Ai Zheng.

[59] Sha D, He YJ (2006). Expression and clinical significance of VEGF and its receptors Flt-1 and KDR in nasopharyngeal carcinoma. Ai Zheng.

[60] Krishna SM, James S, Balaram P (2006). Expression of VEGF as prognosticator in primary nasopharyngeal cancer and its relation to EBV status. Virus Res.

[61] Wang Q, Zhao W, Wu G (2009). Valsartan inhibits NPC cell line CNE-2 proliferation and invasion and promotes its sensitivity to radiation. Eur J Cancer Prev.

[62] Zhou HB, Yin YF, Hu Y, Li X, Zou LY, Li YJ (2011). Suppression of vascular endothelial growth factor via siRNA interference modulates the biological behavior of human nasopharyngeal carcinoma cells. Jpn J Radiol.

[63] Chou J, Lin YC, Kim J, You L, Xu Z, He B (2008). Nasopharyngeal carcinoma--review of the molecular mechanisms of tumorigenesis. Head Neck.

[64] Morris MA, Dawson CW, Young LS (2009). Role of the Epstein-Barr virus-encoded latent membrane protein-1, LMP1, in the pathogenesis of nasopharyngeal carcinoma. Future Oncol.

[65] Zheng H, Li LL, Hu DS, Deng XY, Cao Y (2007). Role of Epstein-Barr virus encoded latent membrane protein 1 in the carcinogenesis of nasopharyngeal carcinoma. Cell Mol Immunol.

[66] Hatzivassiliou E, Mosialos G (2002). Cellular signaling pathways engaged by the Epstein-Barr virus transforming protein LMP1. Front Biosci.

[67] Horikawa T, Yang J, Kondo S, Yoshizaki T, Joab I, Furukawa M (2007). Twist and epithelial-mesenchymal transition are induced by the EBV oncoprotein latent membrane protein 1 and are associated with metastatic nasopharyngeal carcinoma. Cancer Res.

[68] Dawson CW, Laverick L, Morris MA, Tramoutanis G, Young LS (2008). Epstein-Barr virus-encoded LMP1 regulates epithelial cell motility and invasion via the ERK-MAPK pathway. J Virol.

[69] Yang L, Lu Z, Ma X, Cao Y, Sun LQ (2010). A therapeutic approach to nasopharyngeal carcinomas by DNAzymes targeting EBV LMP-1 gene. Molecules.

[70] Ma X, Yang L, Xiao L, Tang M, Liu L, Li Z (2011). Down-regulation of EBV-LMP1 radio-sensitizes nasal pharyngeal carcinoma cells via NF-κB regulated ATM expression. PLoS One.

[71] Xiao L, Hu ZY, Dong X, Tan Z, Li W, Tang M (2014). Targeting Epstein-Barr virus oncoprotein LMP1-mediated glycolysis sensitizes nasopharyngeal carcinoma to radiation therapy. Oncogene.

[72] Litchfield DW (2003). Protein kinase CK2: structure, regulation and role in cellular decisions of life and death. Biochem J.

[73] Liu L, Zou JJ, Luo HS, Wu DH (2009). Effect of protein kinase CK2 gene silencing on radiosensitization in human nasopharyngeal carcinoma cells. Nan Fang Yi Ke Da Xue Xue Bao.

[74] Wang X, Wang Y, Kim HP, Nakahira K, Ryter SW, Choi AM (2007). Carbon monoxide protects against hyperoxia-induced endothelial cell apoptosis by inhibiting reactive oxygen species formation. J Biol Chem.

[75] Deng WT, Wang SL (2010). Research of the relationship between p53, p16, bcl-2, COX-2 and nasopharyngeal carcinoma. J Practi Med.

[76] Li J, Pan YD, Li ZQ, Guo XT, Li LZ (2010). Relations between the expression of survivin, Bcl-2, nm23-H1 and the standardized uptake value (SUV) of 18F-fluorodeoxyglucose in NPC. Guangdong Med J.

[77] Huang T, Chen MH, Wu MY, Wu XY, Huang WJ, Ou HQ (2004). The Relationship between the Expression of bcl-2 and bax Protein and the Radiosensitivity of Nasopharyngeal Carcinoma. CJCO.

[78] Tian XF, Liu DM, Zhang SH, Li GW (2012). Effect of siRNA-mediated Bcl-2 silencing on the radiosensitivity of nasopharyngeal carcinoma cells. J Practi Med.

[79] Lai EC (2004). Notch signaling: control of cell communication and cell fate. Development.

[80] Purow B (2009). Notch inhibitors as a new tool in the war on cancer: a pathway to watch. Curr Pharm Biotechnol.

[81] Guo S, Liu M, Gonzalez-Perez RR (2011). Role of Notch and its oncogenic signaling crosstalk in breast cancer. Biochim Biophys Acta.

[82] Jiang L, Wu J, Chen Q, Hu X, Li W, Hu G (2011). Notch1 expression is upregulated in glioma and is associated with tumor progression. J Clin Neurosci.

[83] Zhang Y, Peng J, Zhang H, Zhu Y, Wan L, Chen J (2010). Notch1 signaling is activated in cells expressing embryonic stem cell proteins in human primary nasopharyngeal carcinoma. J Otolaryngol Head Neck Surg.

[84] Anderson LJ, Longnecker R (2008). An auto-regulatory loop for EBV LMP2A involves activation of Notch. Virology.

[85] Lin J, Zhang XM, Yang JC, Ye YB, Luo SQ (2010). γ-secretase inhibitor-I enhances radiosensitivity of glioblastoma cell lines by depleting CD133+ tumor cells. Arch Med Res.

[86] Yu S, Zhang R, Liu F, Hu H, Yu S, Wang H (2011). Down-regulation of Notch signaling by a γ-secretase inhibitor enhances the radiosensitivity of nasopharyngeal carcinoma cells. Oncol Rep.

[87] Lee CC, Ho CY (2012). Post-treatment late complications of nasopharyngeal carcinoma. Eur Arch Otorhinolaryngol.

[88] Spalding AC, Lawrence TS (2006). New and emerging radiosensitizers and radioprotectors. Cancer Invest.

[89] Kvols LK (2005). Radiation sensitizers: a selective review of molecules targeting DNA and non-DNA targets. J Nucl Med.

[90] Cooper JS, Ang KK (2005). Concomitant chemotherapy and radiation therapy certainly improves local control. Int J Radiat Oncol Biol Phys.

[91] Zhang L, Yang L, Li JJ, Sun L (2012). Potential use of nucleic acid-based agents in the sensitization of nasopharyngeal carcinoma to radiotherapy. Cancer Lett.

